# Combined interventional and craniotomy surgery for meningioma apoplexy complicated by acute subdural hematoma: a case report and literature review

**DOI:** 10.3389/fsurg.2026.1795464

**Published:** 2026-06-18

**Authors:** Chenglong Li, Ting Kang, Yong Ma, Zhenyu Zhang, Jie Zhou, Zhibiao Cai

**Affiliations:** 1 Department of Neurosurgery, The 940th Hospital of Joint Logistics Support Force of Chinese PLA, Lanzhou, Gansu Province, China; 2 The 940th Hospital of Joint Logistics Support Force of Chinese PLA, Lanzhou, Gansu Province, China

**Keywords:** acute subdural hematoma, angiomatous meningioma, interventional therapy, meningioma apoplexy, surgical treatment

## Abstract

**Background:**

Spontaneous hemorrhage from meningiomas causing intracranial hematomas is rare (1%–2% of cases), and concurrent acute subdural hematoma (ASDH) is even rarer, which is easily misdiagnosed and may lead to inadequate preoperative planning. Combined endovascular-craniotomy hybrid surgery enables precise diagnosis and treatment, serving as an effective approach for complex intracranial lesions.

**Case presentation:**

A 34-year-old married female was admitted with a 4-day history of sudden headache. Cranial CT showed left temporal lobe hemorrhage and left frontoparietotemporal-occipital ASDH; CTA indicated a left temporal mass, subdural hematoma, sigmoid sinus compression, cerebral edema, and tortuous superficial vessels. Emergency hybrid surgery was conducted. Digital subtraction angiography (DSA) first ruled out intracranial vascular malformations and confirmed that the left temporal lesion was supplied by the temporal branch of the left middle meningeal artery (ball-holding sign). Given the tumor's location at the cerebral convexity with a relatively superficial distribution, combined with the potential risk of ischemic complications and aggravated cerebral edema secondary to preoperative endovascular embolization, preoperative embolization of tumor-feeding arteries was therefore omitted. Direct craniotomy was subsequently adopted as the definitive surgical approach. A left subtemporal craniotomy was subsequently performed, achieving gross total resection of the tumor (2 × 3 × 4 cm) and complete evacuation of the hematoma. Postoperative pathology confirmed angiomatous meningioma (WHO Grade Ⅰ) with hemorrhage. The patient recovered well without complications or neurological deficits, and no recurrence was noted at 1-year follow-up.

**Conclusion:**

Combined endovascular-craniotomy hybrid surgery achieves integrated diagnosis and treatment through simultaneous endovascular evaluation and craniotomy, improving the efficiency and accuracy in the management of meningioma-related stroke complicated with ASDH. Preoperative endovascular embolization may be added for large, hypervascular tumors to reduce bleeding, but clinicians should remain vigilant for ischemic risks. Rational application of this hybrid surgery effectively improves patient prognosis.

## Introduction

Meningiomas are the most prevalent benign intracranial tumors, with a 5-year survival rate of approximately 69% (1). However, spontaneous hemorrhage from meningiomas leading to intracranial hematoma is relatively rare, occurring in roughly 1%–2% of patients with meningiomas according to the literature (2). Subarachnoid hemorrhage is the most common manifestation, followed by intraparenchymal hemorrhage. Concurrent acute subdural hematoma (ASDH) is even rarer (3–6). This report describes the clinical presentation, imaging findings, treatment strategy, and prognosis of a patient with meningioma apoplexy complicated by ASDH, in whom postoperative pathology confirmed an angiomatous meningioma.

## Clinical data

**Figure 1:**
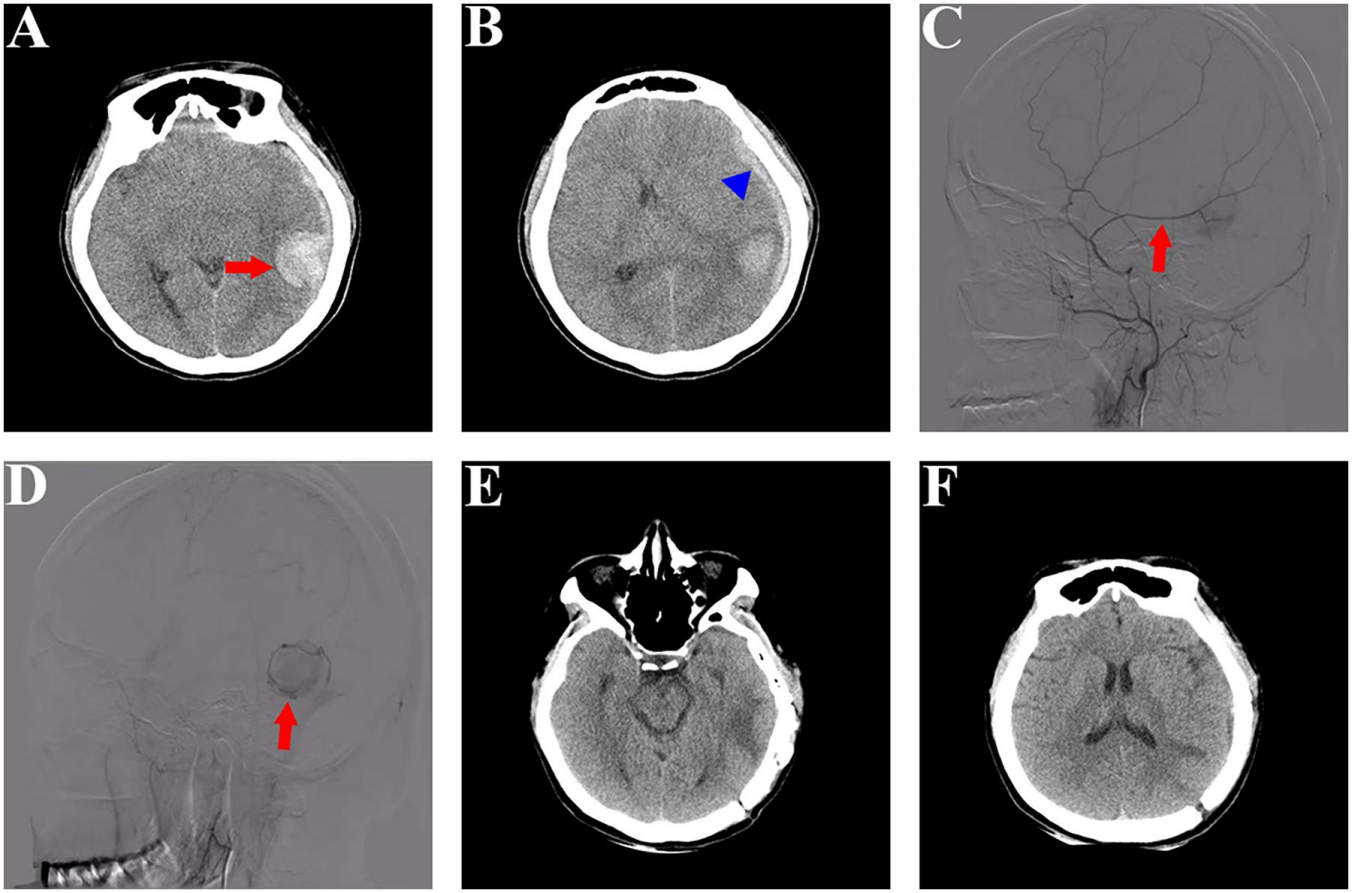
Preoperative CT, intraoperative DSA, and postoperative CT images. **(A,B)** Preoperative head CT showing left temporal lobe hemorrhage and left fronto-parieto-temporal acute subdural hematoma. **(C,D)** Intraoperative DSA demonstrated that the left temporal lesion was supplied by the temporal branch of the left middle meningeal artery, presenting a “ball-holding sign.” **(E,F)** Postoperative head CT confirming complete tumor resection and hematoma evacuation.

A 34-year-old married female presented to the emergency department on March 3, 2025, with a 4-day history of sudden headache. Four days prior to admission, she developed intermittent dull headaches without an obvious trigger, predominantly localized to the left temporal region, which was alleviated by rest. The symptoms were accompanied by dizziness, nausea, and vomiting of gastric contents. Initial non-contrast cranial CT revealed: 1. Intracerebral hemorrhage in the left temporal lobe (approximately 13 mL); 2. Acute subdural hematoma involving the left frontal, parietal, temporal and occipital regions, accompanied by rightward midline shift and subfalcine herniation (Figures 1A,B). Cranio-cervical CTA showed: 1. A slightly hyperdense mass in the left temporal lobe, accompanied by left fronto-parieto-temporal subdural hematoma; 2. Compression and narrowing of the left sigmoid sinus and left transverse sinus compared with the contralateral side; 3. Edema in the left cerebral hemisphere with thickening and tortuosity of superficial vessels. No other abnormalities were detected on cranio-cervical CTA.

Admission examination: Vital signs: Temperature 36.7 °C, pulse 64 beats per minute (bpm), respiratory rate 21 breaths/min, blood pressure 117/75 mmHg, peripheral capillary oxygen saturation (SpO₂) 94%. Neurological examination: The patient was somnolent. Pupils were equal in size, round, and responsive to light (3 mm in diameter). Gross assessment of visual acuity, visual fields, extraocular movements, hearing, and olfaction was normal. Facial symmetry was preserved, the tongue deviated to midline, and the neck was supple. Muscle strength of all limbs was Grade V with normal muscle tone. Reflexes were normal, and no pathological reflexes were elicited. Cardiopulmonary and abdominal examinations revealed no abnormalities. The Glasgow Coma Scale (GCS) score was 13. Admission laboratory tests (complete blood count, biochemistry, infectious disease screening, and coagulation profile) were all within normal ranges.

## Treatment course

Based on the clinical manifestations and imaging findings, the preliminary diagnosis was cerebral tumor apoplexy complicated by ASDH. Intracranial vascular malformation was also considered as a potential cause of hemorrhage. Emergency DSA was performed for further evaluation. DSA revealed a left temporal mass supplied by the temporal branch of the left middle meningeal artery, showing “tumor blush” and vascular encasement (the “ball-holding sign”). The internal carotid artery system did not contribute to the blood supply of the tumor (Figures 1C,D).

**Figure 2:**
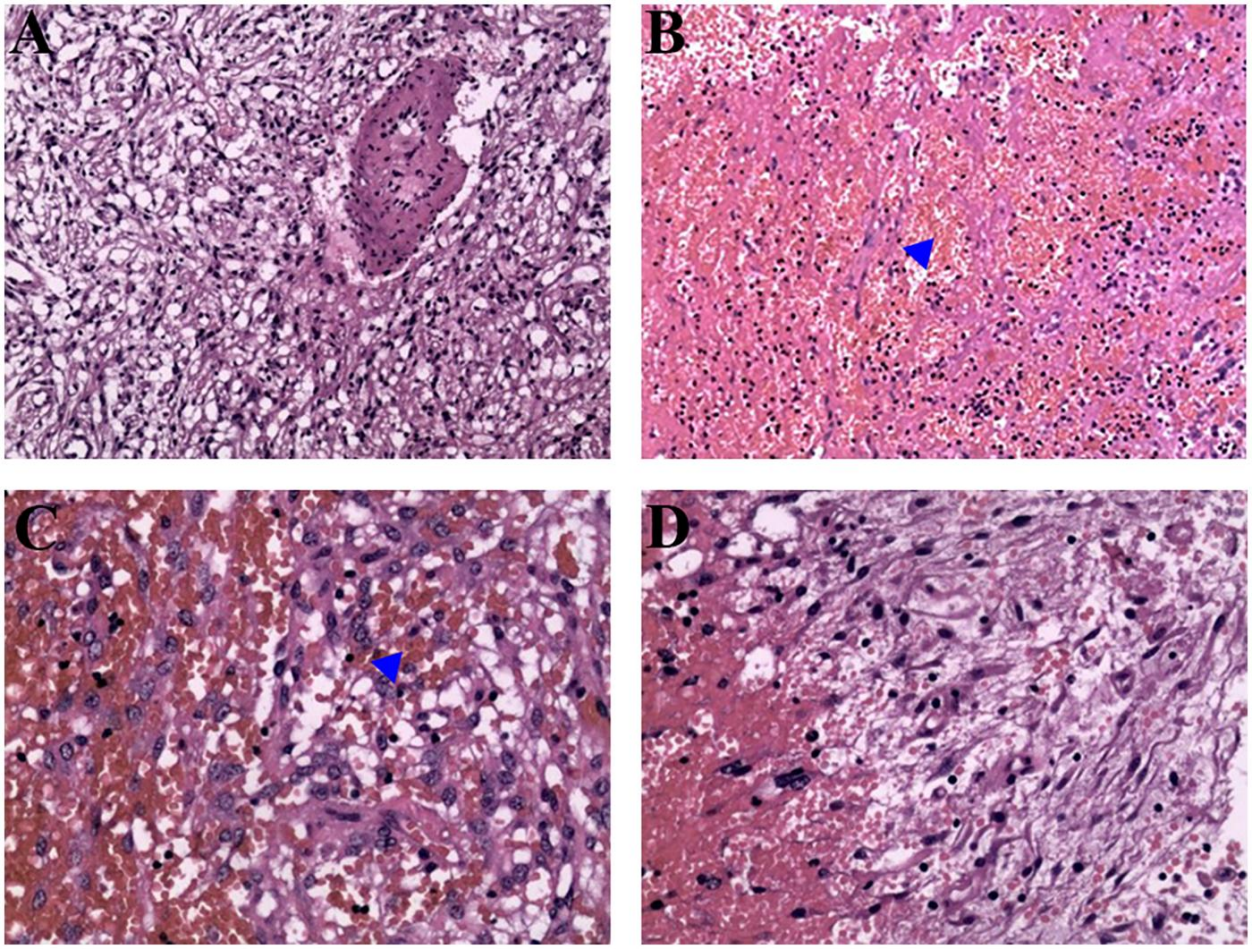
Postoperative pathology confirms angiomatous meningioma, WHO grade I, with intratumoral hemorrhage and hematoma formation. The tumor was composed of spindle cells arranged in fascicles or whorls, with prominent vascularity **(A–D)**.

With vascular malformation excluded by DSA, the diagnosis was confirmed as meningioma apoplexy. Given the tumor's location at the cerebral convexity with a relatively superficial distribution, combined with the potential risk of ischemic complications and aggravated cerebral edema secondary to preoperative endovascular embolization, preoperative embolization of tumor-feeding arteries was therefore omitted. Direct craniotomy was subsequently adopted as the definitive surgical approach. The patient underwent emergency left subtemporal craniotomy under general anesthesia for tumor resection and hematoma evacuation. Intraoperative findings included tense, cyanotic, and highly vascularized dura mater, with compensatory hypertrophy of the middle meningeal artery. Upon dural opening, subdural hematoma and hemorrhagic tumor tissue were visualized. The tumor was densely adherent to the dura mater, dark red in color, of medium consistency, and highly vascular. It was well-demarcated from the surrounding brain parenchyma, measuring approximately 2 × 3 × 4 cm. The tumor was meticulously dissected along the interface with normal brain tissue and completely resected, along with the involved dura mater. Watertight dural closure was performed. The estimated intraoperative blood loss was 400 mL. Postoperative management included hemostatic therapy, dehydration to reduce intracranial pressure, and antiepileptic prophylaxis. Postoperative cranial CT confirmed complete tumor resection and hematoma evacuation, with no complications such as recurrent intracranial hemorrhage (Figures 1E,F). The patient had no postoperative deficits in motor function, speech, or hearing. Pathological report: Angiomatous meningioma (WHO Grade Ⅰ), with intratumoral hemorrhage and hematoma formation. The tumor was composed of spindle cells arranged in fascicles or whorls, with prominent vascularity (Figures 2A–D). Immunohistochemical results: Epithelial Membrane Antigen (EMA) (+), Progesterone Receptor (PR) (+), S100 protein (-), CD34 (-), Nestin (-), Smooth Muscle Actin (SMA) (+), and Ki-67 proliferation index ≈5%. The patient had an uneventful postoperative recovery.

## Discussion

Meningiomas are among the most common intracranial tumors (7). Initial presentation of meningioma as apoplexy complicated by ASDH is extremely rare. In this case, the patient presented with isolated headache without accompanying neurological deficits. Preoperative imaging suggested intracranial hemorrhage with ASDH, and postoperative pathology confirmed angiomatous meningioma apoplexy.

Some scholars have noted that meningiomas presenting with spontaneous hemorrhage are more likely to be misdiagnosed as simple intracranial hemorrhage compared to non-hemorrhagic meningiomas (8). Furthermore, for patients with meningioma apoplexy complicated by ASDH without a clear history of trauma, cerebral angiography is essential to rule out vascular malformations. This not only reduces the risk of misdiagnosis but also facilitates comprehensive treatment planning and improves patient prognosis (9).

The exact pathogenesis of meningioma apoplexy complicated by ASDH remains not fully elucidated. Proposed mechanisms include rupture of abnormal neovascularization within the tumor during growth, stretching and rupture of bridging veins due to tumor mass effect, direct tumor invasion of normal blood vessels, release of vasoactive substances by the tumor, and the use of anticoagulant or antiplatelet agents (3, 4, 10, 11). In this patient, intraoperative findings of abnormally hypertrophied tumor-feeding arteries and compressed draining veins may have contributed to intratumoral hemorrhage and subsequent subdural hematoma formation.

Studies have suggested that risk factors for meningioma apoplexy include age younger than 30 years or older than 70 years, tumor location in the cerebral convexity or ventricles, presence of peritumoral edema, heterogeneous density on CT, and heterogeneous signal with contrast enhancement on magnetic resonance imaging (MRI). Intratumoral cyst formation is considered an independent risk factor for spontaneous hemorrhage in meningiomas (8, 11). In this case, the patient was a 34-year-old female, and preoperative imaging showed a tumor located in the left temporal convexity with peritumoral edema, which may have been potential risk factors for spontaneous hemorrhage.

Treatment options for meningiomas include craniotomy, radiotherapy/chemotherapy, anti-angiogenic therapy, somatostatin analogs, anti-hormonal therapy, and targeted therapy. Currently, complete surgical resection via craniotomy remains the mainstay of treatment (7, 12). For patients with meningioma apoplexy complicated by ASDH, craniotomy is the treatment of choice, as it allows for simultaneous hematoma evacuation and tumor resection. A study on meningioma apoplexy complicated by ASDH reported that among all patients who underwent craniotomy, the mortality rate at a mean follow-up of 3 months was 12%, while 71% of patients were discharged with clinical improvement and no neurological deficits (4). Although most meningiomas are histologically benign, some pose significant surgical challenges due to hypervascularity, deep skull base location, or large size, which can lead to increased intraoperative bleeding and reduced rates of gross total resection. Combined interventional and craniotomy surgery is an important therapeutic strategy for such cases. Preoperative embolization of tumor-feeding arteries via cerebral angiography can reduce tumor vascularity, thereby decreasing intraoperative blood loss, reducing transfusion requirements, and potentially improving the rate of complete resection (10, 13, 14). Multiple studies have demonstrated that, compared with craniotomy alone, combined endoscopic-craniotomy surgery confers significant advantages, including reduced intraoperative blood loss, shorter operative time, a more streamlined surgical workflow, a lower incidence of postoperative hemorrhage, and improved overall patient outcomes (15–17). Furthermore, preoperative embolization does not increase the overall complication rate in patients with meningioma (18). The core value of hybrid endovascular-craniotomy surgery lies in its integration of “diagnosis and treatment.” In emergency settings, this one-stop hybrid procedure—combining angiography, embolization, and tumor resection—confers unique advantages. Specifically, it enables patients to undergo both diagnostic and therapeutic interventions under a single anesthetic session, thereby eliminating the risks of secondary anesthesia, procedural delays, and inter-hospital transport associated with staged surgery, while significantly optimizing the efficiency of emergency care. More importantly, emergency angiography allows for the immediate and accurate exclusion of life-threatening conditions requiring urgent management—including ruptured aneurysms or hemorrhage from cerebral vascular malformations—thus ensuring the accuracy of treatment decision-making and mitigating the risk of misdiagnosis or inappropriate intervention. This facilitates the simultaneous management of hematomas and intracranial tumors in emergency scenarios with enhanced safety and efficacy. However, several studies have indicated that preoperative endovascular embolization of meningioma feeding arteries may increase the incidence of ischemic complications, including postoperative cerebral edema, cerebral infarction, and cerebral venous thrombosis (19, 20). Therefore, careful patient selection for preoperative embolization is crucial, requiring accurate assessment of surgical difficulty and bleeding risk to balance the benefits of reduced hemorrhage and improved resection against the risks of embolization-related complications.

In this case, preoperative DSA ruled out vascular malformations and confirmed the diagnosis of meningioma apoplexy. Although the tumor was hypervascular, its location in the cerebral convexity was relatively superficial. Considering the potential risks of ischemic complications and aggravated edema associated with preoperative embolization, direct craniotomy for complete hematoma evacuation and tumor resection was performed instead of preoperative embolization.

Based on current clinical evidence, the integration of embolization therapy into the management of similar cases is clinically feasible and of substantial potential value. The key is to balance therapeutic benefits and procedural risks via rigorous patient selection and technical optimization. Large retrospective studies have shown that although preoperative embolization fails to improve overall surgical complication rates or resection extent, the degree of tumor embolization acts as an independent predictor of diminished intraoperative blood loss. These findings indicate that enhancing the precision and completeness of embolization techniques could further strengthen its efficacy in reducing intraoperative hemorrhage (16). Moreover, the one-stop hybrid surgical model provides a novel therapeutic direction. This approach enables synchronous embolization and tumor resection within a hybrid operating room. Rather than pursuing complete tumor devascularization, it targets deep feeding arteries that are difficult to expose in the early surgical stage, thereby lowering the risk of tumor edema and other adverse events associated with the waiting period following conventional staged embolization (17). Relevant meta-analyses further support this strategy, confirming that preoperative embolization significantly reduces intraoperative blood loss and operative duration during meningioma resection, without elevating overall complication risks (15). Collectively, optimized embolization strategies can facilitate safer and more radical surgical resection for patients with large, deep-seated, or hypervascular meningiomas.

## Conclusion

In summary, based on this case report and literature review, hemorrhagic meningioma apoplexy is rare and can be easily misdiagnosed as simple intracranial hemorrhage, which may lead to inadequate surgical planning and increased clinical risks. Therefore, for patients with suspected meningioma-related hemorrhage, cerebral angiography is recommended to exclude vascular malformations and delineate the tumor's blood supply, thereby aiding in definitive diagnosis. For large, hypervascular tumors, preoperative embolization of feeding arteries may be considered to reduce intraoperative bleeding. This comprehensive approach can help minimize misdiagnosis and improve outcomes in patients with meningioma apoplexy.

## Data Availability

The original contributions presented in the study are included in the article/Supplementary Material; further inquiries can be directed to the corresponding authors.
